# Super‐stable mineralization effect of layered double hydroxides for heavy metals: Application in soil remediation and perspective

**DOI:** 10.1002/EXP.20210052

**Published:** 2021-10-08

**Authors:** Xianggui Kong, Peipei Hao, Haohong Duan

**Affiliations:** ^1^ State Key Laboratory of Chemical Resource Engineering Beijing University of Chemical Technology Beijing China; ^2^ Department of Chemistry Tsinghua University Beijing China

**Keywords:** contaminated soil, heavy metals, in situ remediation, LDHs, super‐stable mineralization

## Abstract

Agriculture soil plays a crucial role in sustainable development of human society. Unfortunately, soil quality is continuing degradation due to industrial and agricultural activities. Among them, agriculture soil contamination by heavy metals has become a serious threat to global food safety and human health. Because of low‐cost, easy to implement, and fast effects, in situ chemical stabilization strategy has drawn great attention in soil remediation fields. However, since heavy metals are not removed from soil, it is still a great challenge to develop the cost‐effective stabilizers with strong and long‐term immobilization ability. Layered double hydroxides (LDHs) have been extensively applied in environmental fields owing to their unique structure. Very recently, LDHs have been used as amendment in in situ soil remediation for immobilization of heavy metals, exhibiting excellent long‐term stability in practice application through trapping heavy metal ions into the lattice of LDHs layer. Given that the super‐stable mineralization effect of LDHs for heavy metals, we summarize the structure of LDHs, key points of super‐stable mineralization, practical challenges, and potential applications in other heavy metals pollution scenarios in this article, wishing that could provide new strategies and insights into rational designing of amendments for soil remediation.

## Graphical abstract

Owing to the super‐stable mineralization property, layered double hydroxides exhibited excellent performance in soil remediation contaminated by heavy metal ions, providing new strategies and insights into rational designing of amendments in other heavy metals pollution scenarios.

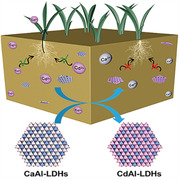

## 1 INTRODUCTION

**FIGURE 1:**
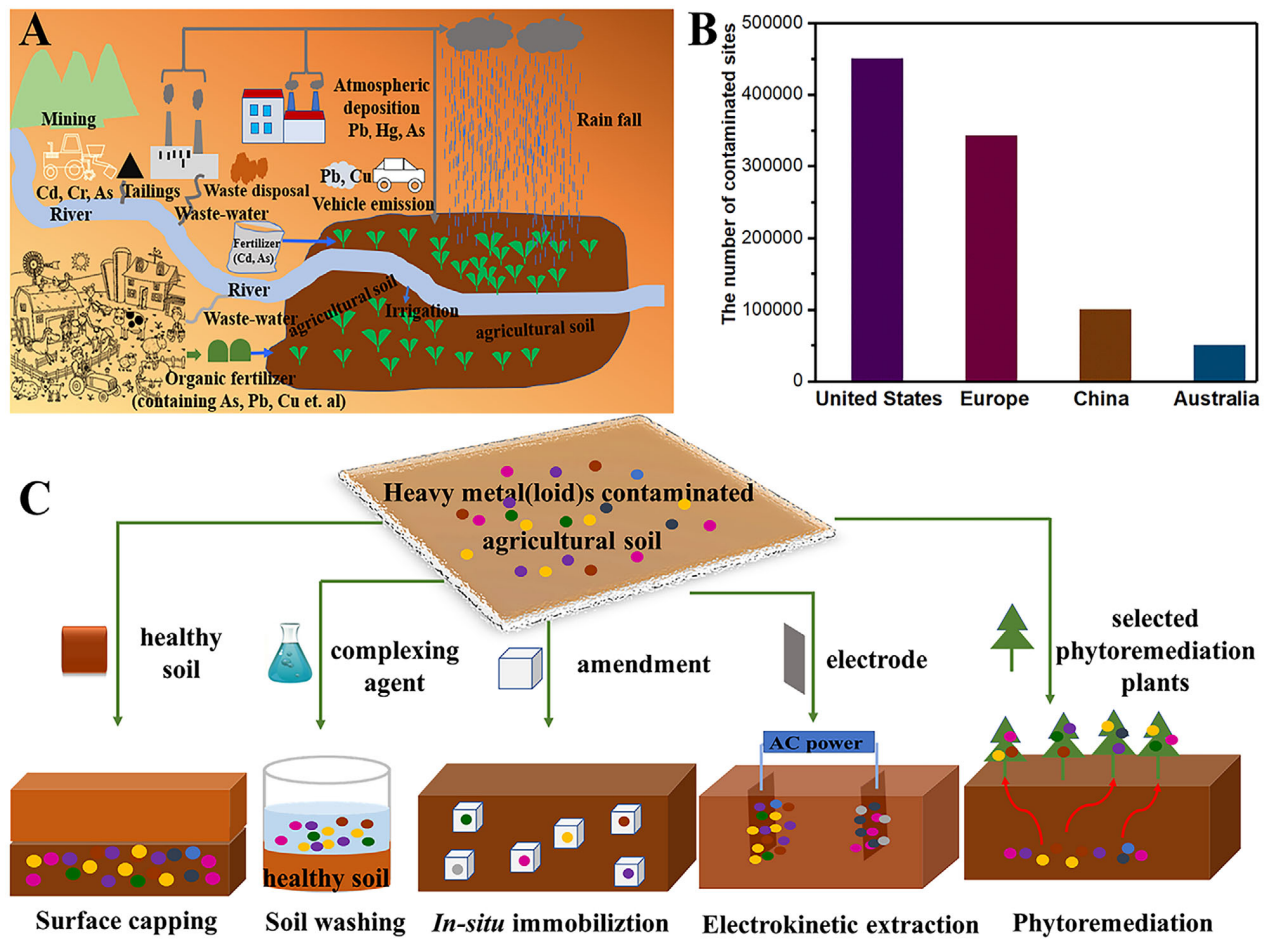
(A) Major sources of heavy metals in agricultural soil; (B) The situation of soil pollution in the word (the dates come from ref. [9]); (C) Illustrated the common remediation techniques for agricultural soil that contaminated by heavy metals

Soil is one of the most vital components for human life on the earth.^[^ ^1^, ^2^ ^]^ However, the quality of soil is continuously exacerbating by amounts of toxic pollutants result from unregulated industry, agricultural activities, and geological changes^[^ ^3^, ^40^ ^]^ (Figure 1A). Among those contaminants, heavy metals are regarded as major hazard to human owing to their nonbiodegradability, carcinogenicity, transferability, and bioaccumulation in the food chain.^[^ ^5^, ^6^, ^7^ ^]^ Soil contamination by heavy metal ions has been a critical global issue and induced a serious threat to human safety and food security in the world^[^ ^8^ ^]^ (Figure 1B). In the United States, there are about 4.5 million pollutant soil sites, in which approximately 600,000 hectare soil contaminated by heavy metals^[^ ^9^ ^]^; and in China, 19% of agricultural soil samples have been polluted by inorganic and/or organic contaminants, in which 82.4% of pollution was caused by heavy metals, and millions of hectares of agricultural soil were taken out of production food due to cadmium (Cd) pollution.^[^ ^10^, ^11^ ^]^In African, it was sad that many children in Nigeria and Senegal died due to Pb‐contaminated soil based on the report of World Health Organization (WHO) in 2018.^[^ ^12^ ^]^ Furthermore, it was reported that the agricultural production in the world must double by 2050 to meet the demand of a growing global population.^[^ ^13^ ^]^ Therefore, it is still an urgent but great challenging work to remediate the pollution soil by heavy metals.

Various soil remediation techniques have sprung up in recent years, which have exhibited different applications depending on the condition of contaminated soil^[^ ^14^, ^15^, ^16^ ^]^ (Figure 1C). Compared with physical and biological remediation methods, chemical remediation method is not only more rapid, cost‐effective under similar remediation goals, but also arouses minimal disturbance to the natural environment by designing well‐directed amendments for the complexity of heavy metal contaminations. Furthermore, the chemical remediation method can reduce the local ecological systems through in situ remediation on large scale.^[^ ^6^ ^]^ Therefore, considering the huge quantity of contaminated agriculture soil, chemical remediation method is a promising remediation technology. Recently, it has been demonstrated that the toxicity of heavy metals in soil is not determined by their total concentrations but is mainly dependent on their bioavailability.^[^ ^17^, ^18^ ^]^ According to this principle, in situ chemical stabilization strategy has been increasingly accepted to remediate heavy metal contaminated soils attributed to its highly efficient, rapid, easy‐to‐apply, and commercial viability.^[^ ^7^, ^8^, ^19^ ^]^ Appropriate amendments are adopted and the heavy metals are treated by surface adsorption, complexation, and/or precipitation. As a result, this in situ chemical stabilization method would retard the transportation and decrease the bioavailability of heavy metals in soil. Although effective and affordable, the heavy metals still exist in the soil by using the in situ chemical stabilization strategy. Therefore, besides capture capability and selectivity, its necessities the amendments have long‐term ability to stabilize the target heavy metals without allowing them to release to the soil again, and excellent stability to adopt complicated natural conditions. Biochar, clay minerals, liming materials, and organic materials are dominant in market.^[^ ^6^ ^]^ However, the sorption capacity and selectivity of biochar and clay minerals for heavy metals were patchy, resulting in a large dosage applied in remediation process. For example, the dosage of palygorskite and sepiolite is recommended at 15–22.5 t/ha for Cd‐contaminated agriculture soil,^[^ ^20^ ^]^ and the dosage of biochar even reaches 50 t/ha.^[^ ^21^ ^]^ Furthermore, owing to the poor affinity to target heavy metals, the immobilized heavy metals are easily remobilized, therefore, considerable dosage of clay minerals and biochar must be repeatedly used for full effectiveness,^[^ ^22^ ^]^ which might cause enrichment of nitrogen and phosphorus with imbalance of crop requirement in agriculture soil.^[^ ^23^ ^]^ Besides, there are multiple heavy metals in contaminated soil, but the remediation performance of clay minerals and biochar varied to different heavy metals. For instance, the bioavailability of Cd apparently decreased while the concentration of Pb increased with biochar as amendment in soil remediation.^[^ ^24^ ^]^ In addition, it must be noted that the biochar derived from waste wood may also contain significant quantities of heavy metals (e.g., Cu, Cr, As, Pb), leading to negative effect on plant growth.^[^ ^21^ ^]^ Liming materials are usually employed as co‐amendments in soil remediation through precipitation the heavy metal ions into carbonate, oxides, or hydroxides. Although the precipitation process is rapid,^[^ ^25^ ^]^ the precipitate was subject to re‐dissolution when the soil pH decreases, resulting in leaching of heavy metal ions. Consequently, there is a pressing need to develop novel amendment with cost‐effective, environmentally friendly, high capture capability and selectivity, durability, and sustainability that can enhance the stabilization performance to target heavy metals in in situ stabilization strategy.

More recently, as a class of anionic clay, layered double hydroxides (LDHs) have shown tremendous promise in fundamental research and practical applications because of their unique layered structure, tunable chemical compositions in host layers, and exchangeable interlayer anions.^[^ ^26^, ^27^, ^28^, ^29^ ^]^ Owing to its high surface area, high layer charge density, and anion exchange capacity, LDHs display excellent adsorption performance to inorganic and organic contaminants in sewage treatment.^[^ ^30^, ^31^, ^32^ ^]^ In the field of soil remediation, as summarized in Table 1, LDHs with different compositions are attracting much more attention as soil amendments for in situ immobilization of heavy metals through ion exchange, adsorption, and precipitation, aiming to decrease the bioavailability and mobility of heavy metals in soil, and thus minimizing the uptake by crops and humans. In this regard, the research is mainly focusing on designing LDH materials to increase the surface adsorption and interlayer anion exchange ability. It is considered that the mineralization of heavy metals into crystals and/or minerals is effective for the in situ stabilization strategy due to its long‐term stability^[^ ^41^, ^42^ ^]^ However, rare research pays attention on stabilizing the heavy metals into the cation layer structure of LDHs.

**TABLE 1:** The application of LDHs for immobilization of heavy metals in soil remediation

**Amendment**	**Target heavy metal ions**	**Immobilization mechanism**	**Remediation performance**	**Ref**.
Single‐layer MgAl‐LDH and CaAl‐LDH	Cr (VI)	Surface adsorption	The leachability of Cr (VI) in soil was reduced by 75.43% and 72.43% with s‐MgAl‐LDH and s‐CaAl‐LDH	^[^ ^33^ ^]^
CaMgFe‐LDH	As (III)	Interlayer ion‐exchange (Mg_2_Fe(OH)_6_(HAsO_3_)) and precipitation (Ca(H_2_AsO_3_)_2_, CaHAsO_4_)	Removal efficiency for As (III) of 47% after 40 days	^[^ ^34^ ^]^
FeAl‐LDH	Cr (VI)	Reduction and precipitation (Cr*_x_* (Fe, Al)_1−_ *_x_* OOH)	The concentration of Cr was decreased from 1477.2 to 646.7 mg/L	^[^ ^35^ ^]^
FeAl‐LDH	Cr (VI)	Surface adsorption, interlayer ion‐exchange (Fe_2_Al(OH)_6_(Cr_2_O_7_)_1/2_), reduction and co‐precipitated with Fe_2_O_3_, Al_2_O_3_, Fe(OH)_3_, and Al(OH)_3_	The immobilization rate reached over 99% at the concentrations of in the soil were 1039 and 2079 mg/kg	^[^ ^36^ ^]^
MgAlFe‐LDH	As, Cu, Cd, Zn	Surface adsorption, precipitation	The immobilizing rates of Cu, Cd, Zn were reached over 95%, and the immobilizing rates of As reached over 90%	^[^ ^37^ ^]^
ZnAl‐LDH	As (III), As (V)	Interlayer ion‐exchange on the external surface and edges, as well as some topotactic exchange	The maximum adsorption capacity of As (III), and As (V) from simulated soil solution was 34.24 and 47.39 mg/g	^[^ ^38^ ^]^
Calcined MgAl‐LDH and CaAl‐LDH	Cr (VI)	Electrostatic and surface adsorption, interlayer ion‐exchange (Mg_4_Al_2_(OH)_12_(HCrO_4_)_2_⋅xH_2_O, Mg_4_Al_2_(OH)_12_CrO_4_⋅yH_2_O)	The immobilizing rates of Cr were 83.54% and 83.33% for MgAl‐LDH (500 ℃) and CaAl‐LDH (900 ℃)	^[^ ^39^ ^]^
MgFe‐LDH and calcined MgFe‐LDH	As (V), Pb (II), and Zn (II)	As: adsorption/incorporation; Pb: adsorption/surface‐induced precipitation; Zn: adsorption, surface‐induced, precipitation, isomorphic substitution	After 10 weeks, the Zn stabilizing potential of LDH and CLDH‐450 reached 99% and 100%; the As stabilizing potential of LDH and CLDH‐450 reached 50% and 14%	^[^ ^40^ ^]^

In this perspective, according to the recently sparked interest in soil remediation with in situ stabilization strategy, we focused on the application of LDHs as amendments for the in situ remediation of heavy metal contaminated soil by mineralization of heavy metals into minerals. The article was structured as follows: The structure of LDHs was introduced in the first section. The second section showed specific examples of heavy metals mineralization into precipitate by using CaAl‐LDHs as the amendment. Lastly, we conclude this perspective by summarizing the key points of super‐stable mineralization and providing potential applications in other heavy metals pollution scenarios.

## 2 THE STRUCTURE OF LDHS

**FIGURE 2:**
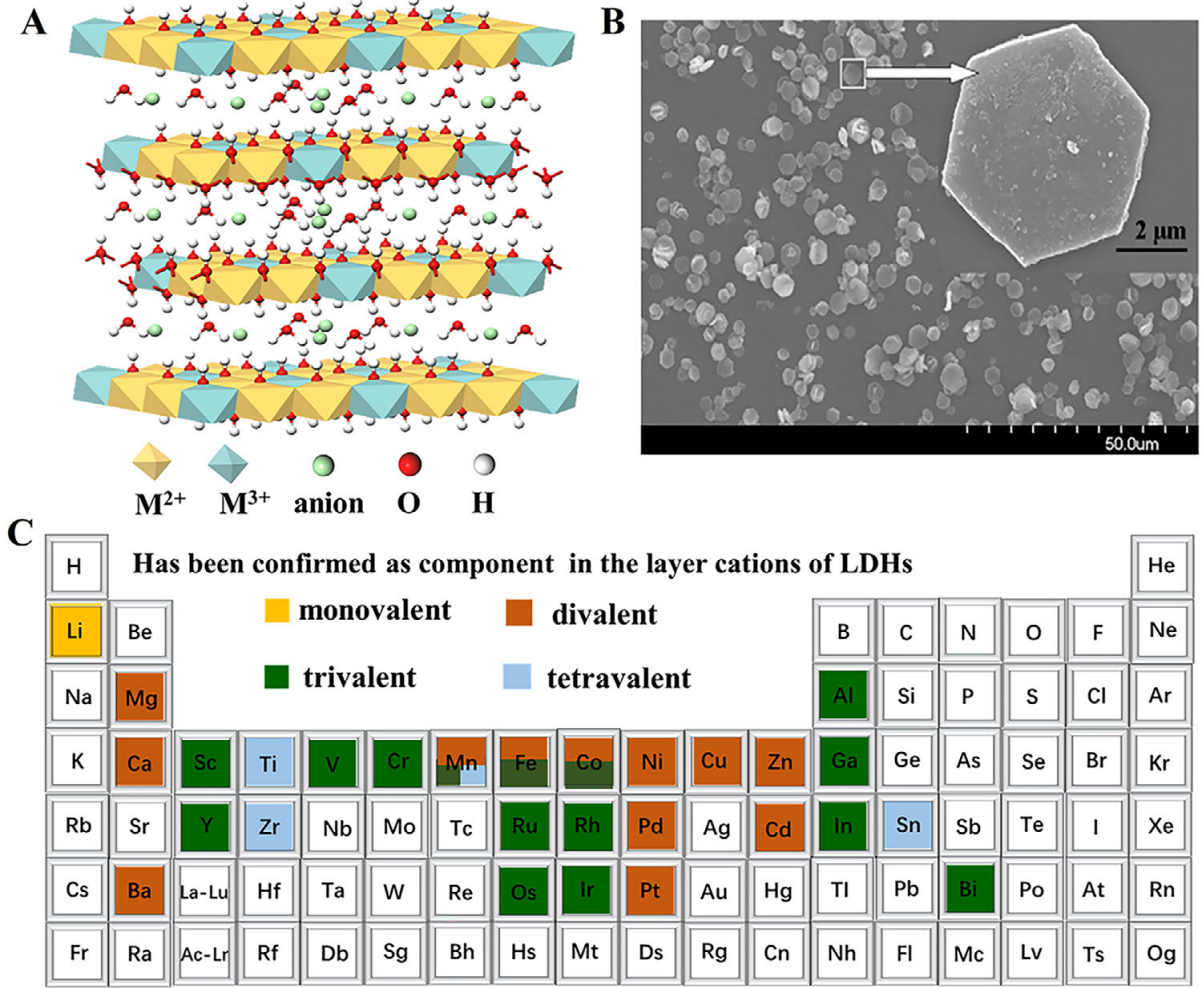
(A) Idealized structure of LDHs; (B) The morphology of Mg_2_Al‐CO_3_‐LDH. Reproduced with permission.^[^ ^29^ ^]^ Copyright 2008, Royal Society of Chemistry; (C) The metal cations that have been reported as components of the layer in LDHs

As a classic host‐guest layered anionic clay, LDHs were firstly discovered in Sweden around 1842 as a natural hydroxycarbonate.^[^ ^43^ ^]^ LDHs have a similar crystal structure with brucite‐like in which the metal cations occupy the centers of edge‐shared octahedra and a fraction of divalent metal cations in layer were replaced by trivalent metal cations resulting in positively charged layers, which in turn lead to anions locating in the interlayer to keep charge neutrality^[^ ^28^, ^29^ ^]^ (Figure 2A). In general, the LDHs have hexagonal nanosheet morphology and the chemical structure formula of LDHs can be descripted as [M^2+^_1−_ *_x_* M^3+^ *_x_* (OH)_2_]^x+^A*^n^* ^–^ *_x_*_/_ *_n_* ·*m*H_2_O, in which M^2+^ and M^3+^ represent divalent and trivalent metal cations, respectively (Figure 2B,C, e.g., Mg^2+^, Ca^2+^, Ni^2+^, Zn^2+^, Al^3+^, Fe^3+^, and Ga^3+^). In addition, the M^2+^ can be substituted by Li^+^ and M^3+^ can be substituted by Ti^4+^, Zr^4+^, and Sn^4+^, respectively.^[^ ^44^, ^45^, ^46^, ^47^ ^]^ A^n–^ is the interlayer anion (e.g., CO_3_^2–^, Cl^–^, NO_3_^–^, SO_4_^2–^, and other inorganic and organic anions), and x represents the molar ratio of M^3+^/(M^2+^+M^3+^) with the value usually ranges from 0.2 to 0.33. The tremendous tunability in types of metal cations, layer charge density, and the interlayer anions results in versatile physical and chemical properties of LDHs, which endows LDHs with promising applications in catalysis,^[^ ^48^, ^49^, ^50^ ^]^ water splitting,^[^ ^51^, ^52^, ^53^, ^54^ ^]^ photochemistry,^[^ ^55^, ^56^, ^57^ ^]^ supercapacitors,^[^ ^58^, ^59^, ^60^ ^]^ adsorption,^[^ ^30^, ^31^, ^32^ ^]^ drug delivery^[^ ^27^ ^]^ and sensors.^[^ ^61^, ^62^ ^]^

As a kind of promising adsorbent for contaminants, LDHs have exhibited excellent adsorption performance to inorganic oxoanions pollutants (e.g., PO_4_^3–^, AsO_4_^–^, HSeO_3_^–^, CrO_4_^2–^),^[^ ^63^, ^64^, ^65^ ^]^ heavy metal ions (Ni^2+^, Cu^2+^, Pb^2+^),^[^ ^66^, ^67^, ^68^ ^]^ radionuclides (e.g., uranium, americium, europium),^[^ ^69^, ^70^, ^71^ ^]^ organic pollutants (e.g., sulfamethoxazole, phenols, azo dyes, tetracycline),^[^ ^31^, ^72^, ^73^ ^]^ and halide ions.^[^ ^74^, ^75^ ^]^ The adsorption mechanism for LDHs to those contaminants mainly involves physical adsorption, electrostatic interaction, anions exchanger, interlayer/surface complexation, and hydroxide precipitation. For example, inspired by the strong covalent bonds between sulfides and heavy metals, Ma and coworkers intercalated MoS_4_^2–^ anions into the gallery of MgAl‐LDH to form MoS_4_‐LDH with excellent adsorption ability to remove various heavy metal ions (Cd^2+^, Hg^2+^, Cu^2+^, Se(VI), etc.)^[^ ^65^, ^66^ ^]^: The concentration of heavy metal ions can be rapidly decreased to extremely low values acceptable for drinking water (ppb level) within a period of several minutes; furthermore, the capture capacity of MoS_4_‐LDH to heavy metals was also markedly elevated (the capture capacity for Se(VI) and Hg^2+^ reached a record value of 294 mg/g and 500 mg/g, respectively). Their results indicated that the intercalated MoS_4_^2–^ played a key role in the adsorption process by formation of M─S bonding (M means heavy metals).

## 3 SUPER‐STABLE MINERALIZATION TO HEAVY METALS

By contrast with water and air contamination, heavy metals in soil can be defined into different fractions based on the sequential extraction procedures.^[^ ^76^, ^77^ ^]^ According to the extensively recognized division, the existence forms of heavy metal in soil were divided into five geochemical fractions by Tessier.^[^ ^78^ ^]^ Among those fractions, heavy metals in the water‐soluble and exchangeable fractions are recognized to be the most toxicity to plants, displaying high bioavailability and mobility to plants. The residual fraction, associated with mineral components, is supposed to represent the least active form, displaying safety to plants. The fractions of carbonate, oxide, and organic components have potential damage to plants under changing conditions. Therefore, the key factor for in situ remediation treatment should be how to transform the heavy metals into residual fraction as much as possible.

Vitrification and thermal treatment are effective methods to immobilize the heavy metals into residual fraction.^[^ ^6^ ^]^ However, the major shortcoming of these treatments is high capital costs and energy consumption. The high temperature requirement in remediation process can cause degradation to soil, inducing the loss of cultivation, and the soil would no longer be able to support agricultural uses.

Besides vitrification and thermal treatment, some researches have found that the heavy metals can be embedded in the crystalline structure of mineral or metal oxides with the assistance of microwave through phase transformation to more stable crystalline mineral, resulting in a remarkable decrement in bioavailability and mobility of heavy metals, and ensuring the long‐term stability and far low leaching concentration of heavy metal ions.^[^ ^79^, ^80^ ^]^ Although this method should not benefit the remediation for farmland soil due to high temperature needed in process, it provides new strategy for the in situ remediation of heavy metal contaminant cropland, namely, the bioavailability and mobility of heavy metal ion can be significantly reduced through anchoring it into the structure of amendment by suitable chemical interaction.

There are multiple kinds of chemical bonding (e.g., interaction between host and guest, hydrogen bond, Van der Waals forces) in the crystal structure of LDHs,^[^ ^29^, ^81^ ^]^ and the metal cations in layer were interconnected with M‐O bonding and M‐M bonding, resulting in a well stability for LDHs. Moreover, the solubility product constant (*K*_sp_) of LDHs is tens orders of magnitude smaller than that of corresponding hydroxides,^[^ ^82^, ^83^ ^]^ indicating that the leachability of metal cations should be remarkably reduced by formation of LDHs from the thermodynamic point of view. Furthermore, LDHs also possesses high buffer capacity in broad range of pH attributed to extensive OH groups on the surface of layer.^[^ ^84^, ^85^ ^]^

**FIGURE 3:**
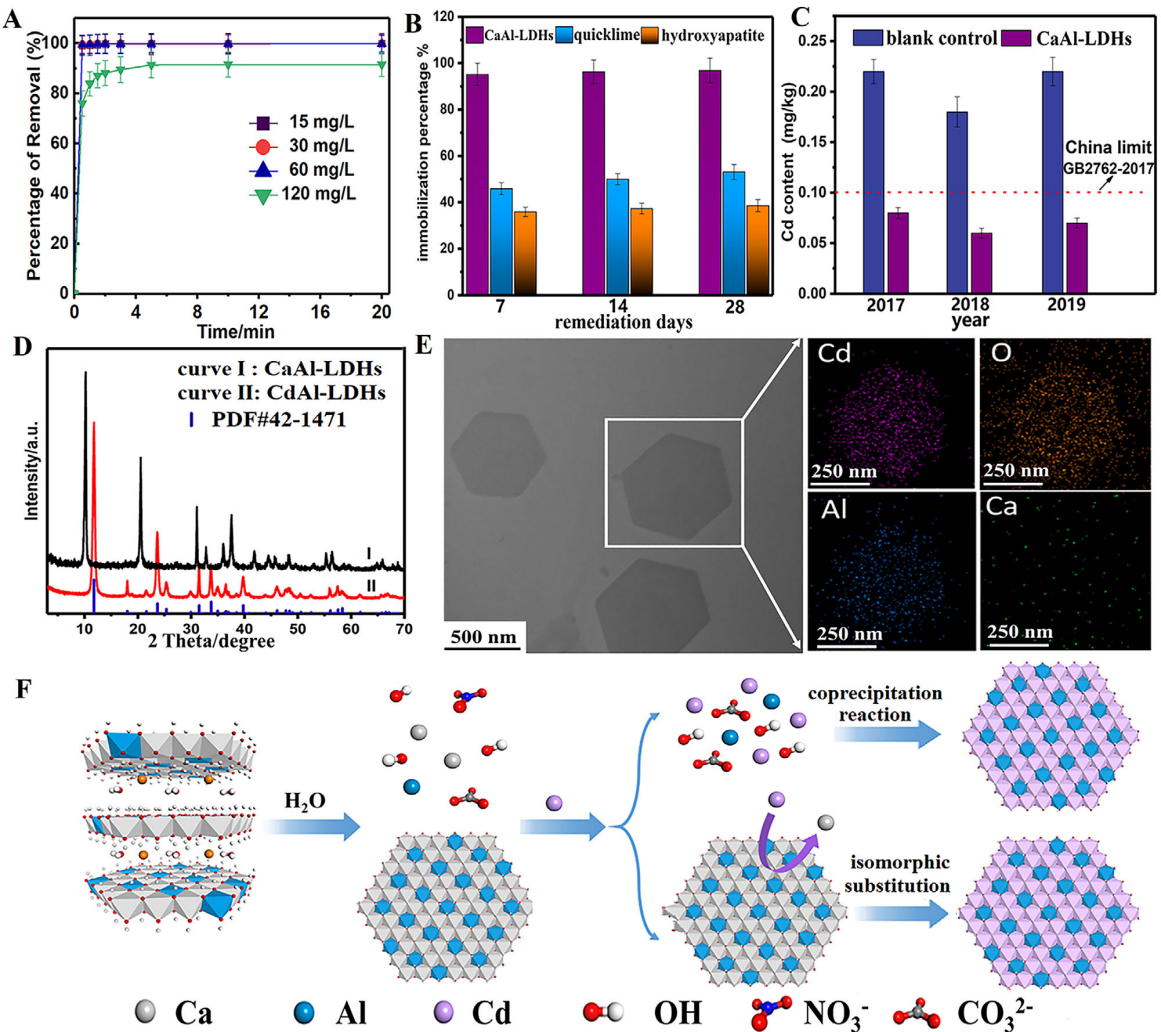
(A) The removal performance of Cd^2+^ at different initial concentrations using CaAl‐LDH in solution system; (B) The immobilization of Cd^2+^ in soil after remediation by CaAl‐LDH, quicklime, and hydroxyapatite; (C) The content of Cd in wheat grain obtained in different years after remediation with CaAl‐LDH as amendment; (D) XRD patterns of samples before and after removal of Cd^2+^; (E) HAADF image and the respective EDS mapping of Cd, Al, O and Ca; (F) Schematic of the proposal mechanism formation of CdAl‐LDH. Reproduced with permission.^[^ ^86^ ^]^ Copyright 2021, Elsevier

Inspired by the advantages of LDHs, in our recent work, we adopted CaAl‐LDH as the amendment for the remediation of Cd‐contaminant soil in lab scale and in practice,^[^ ^86^ ^]^ which displayed excellent performance as follows: The maximum capture capacity for Cd^2+^ reached to 592 mg/g in solution with fast kinetics, surpassing most of the reported adsorbents (Figure 3A); As a soil amendment, the immobilization efficiency for Cd^2+^ reached up to 96.9% in soil remediation within 28 days (Figure 3B), much higher than that of quicklime (53.1%) and hydroxyapatite (23.8%). Furthermore, the practice remediation performance of CaAl‐LDH as the amendment was investigated in the Cd‐contaminated agriculture soil with a scale of 10 ha. Compared with the control group field, the content of Cd in wheat grain obtained from remediation soil is below the standard of China in the first year, and the content of Cd continuously decreased in the followed 2 years (Figure 3C), displaying robust long‐term stability.

According to the experimental results, instead of adsorption on the surface or in the interlayer of CaAl‐LDH, Cd^2+^ was removed in the form of CdAl‐LDH through isomorphic substitution of Ca^2+^ in the layer of CaAl‐LDH (Figure 3D–F). The ultra‐low *K*_sp_ of LDHs caused the heavy metal located in the lattice of layer with long‐term stability, and exhibiting super‐stable mineralization effect in immobilization of heavy metal ions. Besides the mineralization of Cd^2+^, other heavy metal ions such as Ni^2+^, Mn^2+^, and Cu^2+^ also can be mineralized into the formation of LDHs with CaAl‐LDH as mineralizer,^[^ ^87^, ^88^ ^]^ displaying well immobilization performance. Furthermore, the cost of CaAl‐LDH can be cut down to an acceptable price range through technical innovation. For example, CaAl‐LDH can be produced with byproduct of desulphurization gypsum, burnt lime, and aluminum ash as raw materials through adoption of the atom economical technique,^[^ ^87^, ^89^ ^]^ which not only reduced the cost of raw materials but also eradicated the production of wastewater. Therefore, the CaAl‐LDH should be a promising amendment in in situ soil remediation technology.

## 4 SUMMARY AND PERSPECTIVE

Compared with surface adsorption, ion exchange, and surface complexation, it is far advantageous that the heavy metal ions were immobilized through super‐stable mineralization by using LDHs as the mineralizer in in situ soil remediation, which can be summarized as follows:

- High removal capacity to heavy metal ions: Compared with the adsorbents, not only the surface of LDHs, but also the whole LDHs can be used as active site in the mineralization process, leading to high elimination ability to heavy metal ions.
- Fast mineralization rate: Because of the ultra‐low *K*_sp_ of LDHs than that of corresponding carbonate and/or hydroxides, the heavy metal ions can be rapidly mineralized into the formation of LDHs within several minutes, leading to excellent capture efficiency to heavy metal ions.
- Excellent stability: Compared with surface adsorption, the heavy metal ions were embedded into the layer lattice of LDHs with ultra‐low *K*_sp_, suggesting that the leachability of heavy metal ions from LDHs can be significant reduced; furthermore, the LDHs possessed abundant ─OH group in the layer, which can enhance the resistance to acid rain. These properties guaranteed that the heavy metal ions can be anchored in the lattice of LDHs with well stability and long‐term sustainability in soil remediation.
- Well selectivity: Soil is a kind of quite complicated mixture; it is a big challenge to mineralize the heavy metal ions with well selectivity. According to the formation principle of LDHs, only the metal cations with suitable ionic radius, similar coordination structure, and valence can be embedded into the layer lattice of LDHs through isomorphic substitution, therefore, the mineralization selectivity to target heavy metal ions can be improved by choosing suitable LDHs as mineralizer owing to the tunability of LDHs.
- Acceptable price and scaling up production: In view of the huge quantity of contaminated soil by heavy metals, the cost and scaling up production of amendment must be considered in application. With the assistance of technological innovation, a series of LDHs compounds can be produced on large scale through green synthesis method, resulting in low cost and high efficiency.

**FIGURE 4:**
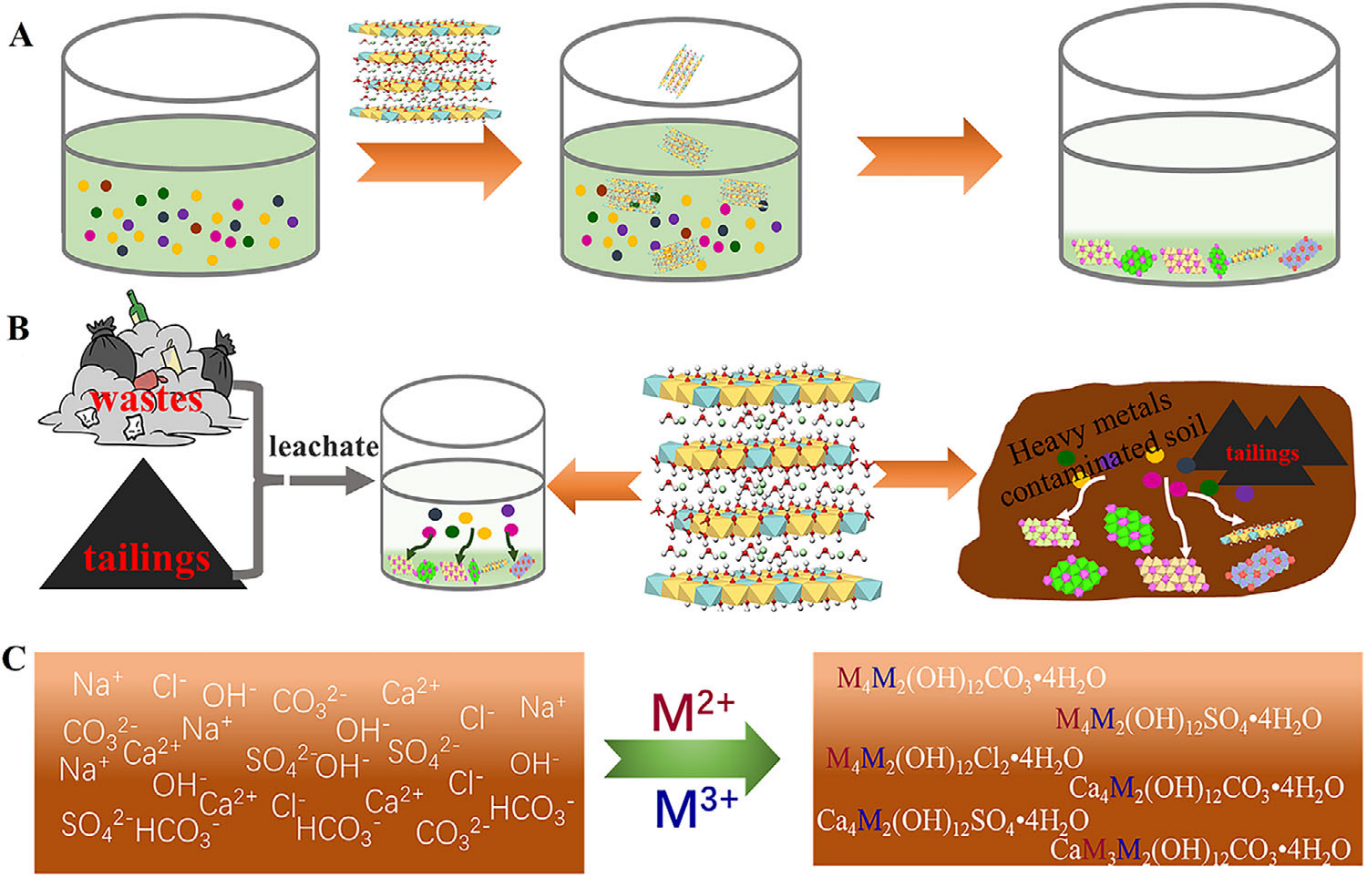
The application of LDHs as amendment to (A) electroplating wastewater, (B) landfill leachate and soil pollutant by tailings; (C) The remediation of saline‐alkali soil with in situ formation LDHs

Based on its well performance to mineralize the heavy metals, LDHs also can be used as amendment in many environmental fields besides soil remediation, especially in the following aspects (Figure 4):

1. Electroplating wastewater: Electroplating industry plays very important role in social development. However, a lot of wastewater contains Ni^2+^, Zn^2+^, Cu^2+^, and Cd^2+^ which results in serious water pollution problems if the wastewater is not well treatment before discharging. Chemical precipitation and coagulation‐flocculation are the main techniques in practical treatment; nevertheless, the main drawbacks are the large amounts of sludge and low efficiency. Therefore, it would be a good choice to remediate the electroplating wastewater with suitable LDHs as the mineralizer through mineralization the heavy metal ions into LDHs form, owing to its high capacity, fast removal rate, and good selectivity to target ions. Furthermore, the products obtained from the mineralization might be used as electrocatalytic materials (NiAl‐LDH), photocatalytic materials (ZnCr‐LDH), and catalyst supports (CuAl‐LDH). In other words, with the aid of super‐stable mineralization effect of LDHs for heavy metals, the heavy metal ions in electroplating wastewater not only can be efficiently disposed, but the final products may also reuse as functional materials for other applications.
2. Contaminated sites and landfill leachate: There are a lot of heavy metal ions in contaminated sites and landfill leachate, which also can cause serious pollution to water and/or soil. Similar to soil remediation, LDHs can also be used in these fields as promising amendment to realize the in situ mineralization of heavy metal ions.
3. Saline‐alkali soil remediation: The salinization and alkalization of soil is another severe problem that threatens the food security of human life, which accounts for about 10% of the agriculture soil in the world. According to the mechanism of super‐stable mineralization, the anions of SO_4_^2–^, CO_3_^2–^, and OH^–^ in saline‐alkali soil are the necessary elements for the formation of LDHs. Therefore, if some suitable metal cations are added into the soil, the anions of SO_4_^2–^, CO_3_^2–^, and OH^–^ should be quickly immobilized by formation of LDHs, giving rise to decreasing of salinization and alkalization of soil.

As featured in this perspective, LDHs have been exhibited outstanding performance in in situ immobilization of heavy metal ions. However, there are several challenges need to be overcome in future. First, although some LDHs (e.g., MgAl‐LDH, CaAl‐LDH) have been produced by atom‐economic synthesis methods, these methods are still difficult to expand to a broad selection of LDHs. Therefore, a general method to produce LDHs with green technology is yet to be developed, in order to reduce even eliminate the byproduct such as NaCl, Na_2_SO_4_, or/and NaNO_3_ in the fabrication process; Second, although the LDHs displays high performance of removal heavy metal ions in sewage treatment, it is difficult to achieve the solid‐liquid separation in practice application with simple‐to‐use and low‐energy, because LDHs remain to suspend stably in suspension after remove of heavy metals due to their nano/micrometer size. Thus, special attention in the future should be paid to the formation of LDHs with suitable size or morphology that can be quickly separated from solution by gravity sedimentation; Third, owing to the different sources of heavy metals, the contaminated soil usually contains multiple heavy metals with different component and proportion, therefore, a single kind of LDHs will have difficulty to mineralize all of the heavy metals in remediation process, it is necessary to design various types of LDHs amendments for different contaminated soil. Furthermore, soil is a mixture of minerals, water, air, and living organisms, giving rise to a much more complex environment; it is critical to clarify the mineralization mechanisms for heavy metal ions in field practices, which can be considered as one key step to achieve breakthroughs in in situ soil remediation.

Overall, LDHs have been demonstrated to be a promising amendment for heavy metal ions polluted soil due to its tunable structure and intrinsic physical and chemical properties, given that some of them have already been used in practice remediation for Cd‐contaminated agriculture soil based on super‐stable mineralization. Apart from the application in remediation of contaminated soil by heavy metals, the remarkable super‐stable mineralization performances to heavy metals endow LDHs with great potential application in the remediation of electroplating wastewater, landfill leachate, and the improvement of saline‐alkali soil. Although LDHs material has displayed excellent performance in soil remediation, there is still much room for improvements in chemical composition, morphology, particle size, and optimization of production strategy. With this perspective, as we believe, which could provide new strategies and insights into rational designing of amendments for soil remediation.

## CONFLICT OF INTEREST

The authors declare that they have no known competing financial interests or personal relationships that could have appeared to influence the work reported in this paper.
